# 3-Benzoyl-4-hydr­oxy-2-methyl-2*H*-1,2-benzothia­zine 1,1-dioxide

**DOI:** 10.1107/S1600536810011025

**Published:** 2010-03-27

**Authors:** Matloob Ahmad, Hamid Latif Siddiqui, Saeed Ahmad, Sana Aslam, Masood Parvez

**Affiliations:** a Applied Chemistry Research Centre, PCSIR Laboratories Complex, Lahore-54600, Pakistan; b Institute of Chemistry, University of the Punjab, Lahore 54590, Pakistan; c Department of Chemistry, Gomal University, Dera Ismail Khan, NWFP, Pakistan; d Department of Chemistry, The University of Calgary, 2500 University Drive NW, Calgary, Alberta, Canada T2N 1N4

## Abstract

In the title mol­ecule, C_16_H_13_NO_4_S, the heterocyclic thia­zine ring adopts a half-chair conformation with the S and N atoms displaced by 0.410 (3) and 0.299 (3) Å, respectively, on opposite sides of the mean plane formed by the remaining ring atoms. The crystal structure is stabilized by inter­molecular hydrogen bonds of the types O—H⋯O and C—H⋯O; the former result in dimers lying about inversion centers and the latter form chains of mol­ecules running along the *c* axis. In addition, intra­molecular O—H⋯O links are present.

## Related literature

For 1,2-benzothia­zine derivatives as anti-inflammatory drugs (NSAIDs), see: Lombardino *et al.* (1971 ▶); Soler (1985 ▶); Carty *et al.* (1993 ▶); Turck *et al.* (1995 ▶). For the synthesis of benzothia­zine derivatives, see: Siddiqui *et al.* (2007 ▶); Ahmad, Siddiqui, Zia-ur-Rehman *et al.* (2010 ▶). For related structures, see: Siddiqui *et al.* (2008 ▶); Ahmad, Siddiqui, Rizvi *et al.* (2010 ▶).
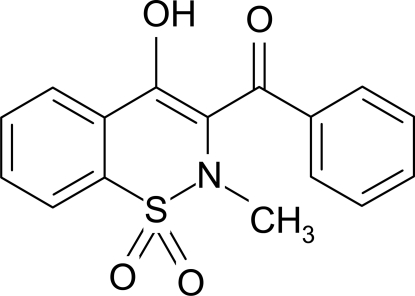


## Experimental

### 

#### Crystal data

C_16_H_13_NO_4_S
*M* *_r_* = 315.33
Triclinic, 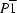

*a* = 6.8342 (3) Å
*b* = 9.9085 (3) Å
*c* = 10.7234 (4) Å
α = 83.257 (2)°
β = 79.481 (2)°
γ = 85.113 (2)°
*V* = 707.50 (5) Å^3^
*Z* = 2
Mo *K*α radiation
μ = 0.25 mm^−1^
*T* = 173 K
0.12 × 0.10 × 0.08 mm

#### Data collection

Nonius KappaCCD diffractometer
Absorption correction: multi-scan (*SORTAV*; Blessing, 1997 ▶) *T*_min_ = 0.971, *T*_max_ = 0.981
7177 measured reflections
4080 independent reflections
3665 reflections with *I* > 2σ(*I*)
*R*_int_ = 0.022

#### Refinement

*R*[*F* ^2^ > 2σ(*F* ^2^)] = 0.041
*wR*(*F* ^2^) = 0.113
*S* = 1.09
4080 reflections
201 parameters
H-atom parameters constrained
Δρ_max_ = 0.42 e Å^−3^
Δρ_min_ = −0.34 e Å^−3^

### 

Data collection: *COLLECT* (Hooft, 1998 ▶); cell refinement: *DENZO* (Otwinowski & Minor, 1997 ▶); data reduction: *SCALEPACK* (Otwinowski & Minor, 1997 ▶); program(s) used to solve structure: *SHELXS97* (Sheldrick, 2008 ▶); program(s) used to refine structure: *SHELXL97* (Sheldrick, 2008 ▶); molecular graphics: *ORTEP-3 for Windows* (Farrugia, 1997 ▶); software used to prepare material for publication: *SHELXL97*.

## Supplementary Material

Crystal structure: contains datablocks global, I. DOI: 10.1107/S1600536810011025/jh2140sup1.cif

Structure factors: contains datablocks I. DOI: 10.1107/S1600536810011025/jh2140Isup2.hkl

Additional supplementary materials:  crystallographic information; 3D view; checkCIF report

## Figures and Tables

**Table 1:** Hydrogen-bond geometry (Å, °)

*D*—H⋯*A*	*D*—H	H⋯*A*	*D*⋯*A*	*D*—H⋯*A*
O3—H3*O*⋯O4	0.84	1.80	2.5365 (15)	146
O3—H3*O*⋯O1^i^	0.84	2.56	3.0108 (15)	115
C3—H3⋯O1^ii^	0.95	2.50	3.2627 (18)	138

Symmetry codes: (i) 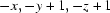; (ii) 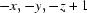.

**Fig. 1.**
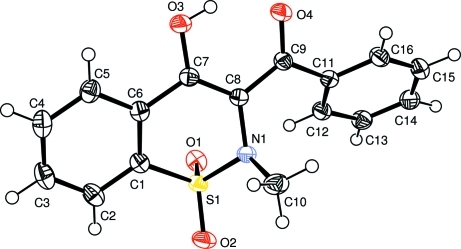
The title molecule plotted with the displacement ellipsoids at 50% probability level (Farrugia, 1997).

**Fig. 2.**
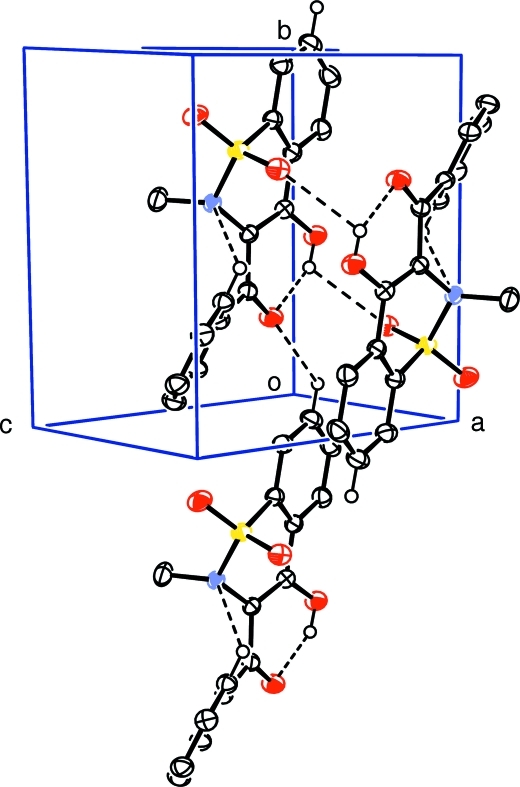
A part of the unit cell showing intermolecular and intrmolecular hydrogen bonds by dashed lines; the H-atoms not involved in H-bonds have been excluded for clarity.

## supplementary crystallographic information

### Comment

Oxicam, a class of non steroidal anti-inflammatory drugs (NSAIDs) consists of benzothiazine derivatives which are found to be potent anti-inflammatory and analgesic agents, e.g., piroxicam (Lombardino *et al.*, 1971), droxicam (Soler, 1985), ampiroxicam (Carty *et al.*, 1993), meloxicam (Turck *et al.*, 1995), etc. In continuation of our research on potential biologically active benzothiazine compounds (Siddiqui *et al.*, 2007; Ahmad, Siddiqui, Zia-ur-Rehman *et al.*, 2010), we report the synthesis and crystal structure of the title compound in this article.

In the title compound (Fig. 1), the bond distances and angles agree with the cortresponding bond distances and angles reported in closely related compounds (Siddiqui *et al.*, 2008; Ahmad, Siddiqui, Rizvi *et al.*, 2010). The heterocyclic thiazine ring adopts half chair conformation with atoms S1 and N1 displaced by 0.410 (3) and 0.299 (3) Å on the opposite sides from the mean planes formed by the remaining ring atoms.

The structure is stabilized by intermolecular hydrogen bonds of the types O—H···O and C—H···O; the former result in dimers lying about inversion centers and the later form chains of molecules running along the *c*-axis (Tab. 1 and Fig. 2). In addition, intramolecular interactions of the types O—H···O and C—H···N are also present consolidating the crystal packing.

### Experimental

An aqueous sodium hydroxide solution (1.33 g in 10 ml water) was slowly added to a solution of 3-benzoyl-4-hydroxy-2*H*-1,2-benzothiazine 1,1-dioxide (5.0 g, 16.6 mmole) in acetone (50 ml). Dimethylsulfate (4.03 g, 32 mmole) was added drop wise and the mixture was stirred for 30 minutes. The contents of the flask were acidified to pH 3.0 by the addition of 5% HCl. White precipitates of the title compound were formed which were collected and washed with excess distilled water. Crystals suitable for crystallographic study were grown from a solution of chloroform/methanol (4:1); yield = 3.5 g, 70%; m.p. = 420-421 K.

### Refinement

Though all the H atoms could be distinguished in the difference Fourier map the H-atoms were included at geometrically idealized positions and refined in riding-model approximation with O—H = 0.84 Å and C—H = 0.95 and 0.98 Å for aryl and methyl H-atoms, respectively. The *U*_iso_(H) were allowed at 1.2*U*_eq_(O/C). The final difference map was essentially featurless.

### Crystal data

**Table:**

C_16_H_13_NO_4_S	*Z* = 2
*M**_r_* = 315.33	*F*(000) = 328
Triclinic, *P*1	*D*_x_ = 1.480 Mg m^−^^3^
Hall symbol: -P 1	Mo *K*α radiation, λ = 0.71073 Å
*a* = 6.8342 (3) Å	Cell parameters from 3532 reflections
*b* = 9.9085 (3) Å	θ = 1.0–30.0°
*c* = 10.7234 (4) Å	µ = 0.25 mm^−^^1^
α = 83.257 (2)°	*T* = 173 K
β = 79.481 (2)°	Block, pale-yellow
γ = 85.113 (2)°	0.12 × 0.10 × 0.08 mm
*V* = 707.50 (5) Å^3^	

### Data collection

**Table:**

Nonius KappaCCD diffractometer	4080 independent reflections
Radiation source: fine-focus sealed tube	3665 reflections with *I* > 2σ(*I*)
graphite	*R*_int_ = 0.022
ω and φ scans	θ_max_ = 30.1°, θ_min_ = 2.1°
Absorption correction: multi-scan (*SORTAV*; Blessing, 1997)	*h* = −9→9
*T*_min_ = 0.971, *T*_max_ = 0.981	*k* = −13→13
7177 measured reflections	*l* = −15→15

### Refinement

**Table:**

Refinement on *F*^2^	Primary atom site location: structure-invariant direct methods
Least-squares matrix: full	Secondary atom site location: difference Fourier map
*R*[*F*^2^ > 2σ(*F*^2^)] = 0.041	Hydrogen site location: inferred from neighbouring sites
*wR*(*F*^2^) = 0.113	H-atom parameters constrained
*S* = 1.09	*w* = 1/[σ^2^(*F*_o_^2^) + (0.0468*P*)^2^ + 0.3608*P*] where *P* = (*F*_o_^2^ + 2*F*_c_^2^)/3
4080 reflections	(Δ/σ)_max_ < 0.001
201 parameters	Δρ_max_ = 0.42 e Å^−^^3^
0 restraints	Δρ_min_ = −0.34 e Å^−^^3^

### Special details

**Table:**

Geometry. All esds (except the esd in the dihedral angle between two l.s. planes) are estimated using the full covariance matrix. The cell esds are taken into account individually in the estimation of esds in distances, angles and torsion angles; correlations between esds in cell parameters are only used when they are defined by crystal symmetry. An approximate (isotropic) treatment of cell esds is used for estimating esds involving l.s. planes.
Refinement. Refinement of *F*^2^ against ALL reflections. The weighted *R*-factor wR and goodness of fit *S* are based on *F*^2^, conventional *R*-factors *R* are based on *F*, with *F* set to zero for negative *F*^2^. The threshold expression of *F*^2^ > σ(*F*^2^) is used only for calculating *R*-factors(gt) etc. and is not relevant to the choice of reflections for refinement. *R*-factors based on *F*^2^ are statistically about twice as large as those based on *F*, and *R*- factors based on ALL data will be even larger.

### Fractional atomic coordinates and isotropic or equivalent isotropic displacement parameters (Å^2^)

**Table:**

	*x*	*y*	*z*	*U*_iso_*/*U*_eq_	
S1	0.20282 (5)	0.24470 (3)	0.28051 (3)	0.02305 (10)	
O1	−0.00548 (16)	0.28521 (10)	0.28975 (11)	0.0279 (2)	
O2	0.29460 (19)	0.15793 (11)	0.18540 (11)	0.0339 (3)	
O3	0.23671 (18)	0.47875 (11)	0.58638 (10)	0.0290 (2)	
H3O	0.2333	0.5621	0.5604	0.044*	
O4	0.24683 (18)	0.68733 (10)	0.42430 (10)	0.0303 (2)	
N1	0.32262 (17)	0.38365 (11)	0.25903 (11)	0.0218 (2)	
C1	0.2435 (2)	0.17087 (13)	0.43178 (14)	0.0230 (3)	
C2	0.2400 (2)	0.03080 (14)	0.46264 (16)	0.0284 (3)	
H2	0.2244	−0.0268	0.4008	0.034*	
C3	0.2596 (2)	−0.02341 (15)	0.58521 (17)	0.0321 (3)	
H3	0.2570	−0.1189	0.6078	0.039*	
C4	0.2832 (2)	0.06112 (16)	0.67508 (16)	0.0325 (3)	
H4	0.2965	0.0229	0.7588	0.039*	
C5	0.2875 (2)	0.20118 (15)	0.64393 (14)	0.0279 (3)	
H5	0.3035	0.2582	0.7062	0.033*	
C6	0.2683 (2)	0.25797 (13)	0.52084 (13)	0.0228 (3)	
C7	0.2650 (2)	0.40639 (13)	0.48734 (13)	0.0218 (2)	
C8	0.2777 (2)	0.46608 (13)	0.36361 (13)	0.0205 (2)	
C9	0.2466 (2)	0.61275 (13)	0.33796 (13)	0.0222 (2)	
C10	0.5305 (2)	0.38032 (17)	0.19048 (16)	0.0336 (3)	
H10A	0.5688	0.4736	0.1624	0.040*	
H10B	0.5419	0.3296	0.1161	0.040*	
H10C	0.6189	0.3356	0.2474	0.040*	
C11	0.2056 (2)	0.67762 (13)	0.21160 (13)	0.0227 (3)	
C12	0.0803 (2)	0.61998 (14)	0.14609 (14)	0.0254 (3)	
H12	0.0330	0.5325	0.1758	0.030*	
C13	0.0252 (2)	0.69159 (15)	0.03694 (14)	0.0283 (3)	
H13	−0.0619	0.6535	−0.0071	0.034*	
C14	0.0968 (3)	0.81858 (15)	−0.00811 (15)	0.0304 (3)	
H14	0.0587	0.8668	−0.0828	0.036*	
C15	0.2239 (3)	0.87500 (15)	0.05592 (15)	0.0309 (3)	
H15	0.2752	0.9609	0.0240	0.037*	
C16	0.2759 (2)	0.80577 (14)	0.16662 (14)	0.0270 (3)	
H16	0.3595	0.8456	0.2119	0.032*	

### Atomic displacement parameters (Å^2^)

**Table:**

	*U*^11^	*U*^22^	*U*^33^	*U*^12^	*U*^13^	*U*^23^
S1	0.02533 (17)	0.02010 (16)	0.02556 (17)	−0.00128 (11)	−0.00723 (12)	−0.00570 (11)
O1	0.0246 (5)	0.0277 (5)	0.0338 (6)	−0.0033 (4)	−0.0113 (4)	−0.0022 (4)
O2	0.0448 (7)	0.0258 (5)	0.0331 (6)	0.0001 (4)	−0.0074 (5)	−0.0127 (4)
O3	0.0404 (6)	0.0251 (5)	0.0231 (5)	0.0006 (4)	−0.0084 (4)	−0.0063 (4)
O4	0.0444 (6)	0.0224 (5)	0.0263 (5)	−0.0038 (4)	−0.0088 (4)	−0.0065 (4)
N1	0.0218 (5)	0.0210 (5)	0.0224 (5)	−0.0014 (4)	−0.0020 (4)	−0.0053 (4)
C1	0.0189 (6)	0.0221 (6)	0.0281 (7)	−0.0007 (4)	−0.0050 (5)	−0.0017 (5)
C2	0.0218 (6)	0.0220 (6)	0.0412 (8)	−0.0019 (5)	−0.0061 (6)	−0.0017 (5)
C3	0.0234 (7)	0.0245 (6)	0.0460 (9)	−0.0031 (5)	−0.0055 (6)	0.0068 (6)
C4	0.0278 (7)	0.0326 (7)	0.0337 (8)	−0.0019 (6)	−0.0045 (6)	0.0093 (6)
C5	0.0261 (7)	0.0311 (7)	0.0256 (7)	−0.0004 (5)	−0.0053 (5)	0.0012 (5)
C6	0.0194 (6)	0.0230 (6)	0.0254 (6)	−0.0004 (4)	−0.0040 (5)	−0.0010 (5)
C7	0.0212 (6)	0.0226 (6)	0.0226 (6)	−0.0010 (4)	−0.0054 (5)	−0.0038 (5)
C8	0.0209 (6)	0.0202 (5)	0.0211 (6)	−0.0011 (4)	−0.0041 (4)	−0.0042 (4)
C9	0.0227 (6)	0.0211 (6)	0.0234 (6)	−0.0033 (4)	−0.0039 (5)	−0.0032 (4)
C10	0.0259 (7)	0.0364 (8)	0.0373 (8)	−0.0034 (6)	0.0036 (6)	−0.0123 (6)
C11	0.0255 (6)	0.0200 (6)	0.0222 (6)	0.0002 (5)	−0.0033 (5)	−0.0033 (4)
C12	0.0278 (7)	0.0232 (6)	0.0258 (6)	−0.0021 (5)	−0.0054 (5)	−0.0036 (5)
C13	0.0319 (7)	0.0287 (7)	0.0261 (7)	−0.0005 (5)	−0.0093 (6)	−0.0046 (5)
C14	0.0391 (8)	0.0266 (7)	0.0249 (7)	0.0036 (6)	−0.0077 (6)	−0.0012 (5)
C15	0.0404 (8)	0.0216 (6)	0.0301 (7)	−0.0031 (5)	−0.0058 (6)	0.0005 (5)
C16	0.0312 (7)	0.0217 (6)	0.0294 (7)	−0.0034 (5)	−0.0070 (6)	−0.0036 (5)

### Geometric parameters (Å, °)

**Table:**

S1—O2	1.4329 (11)	C6—C7	1.4716 (18)
S1—O1	1.4346 (11)	C7—C8	1.3784 (19)
S1—N1	1.6333 (12)	C8—C9	1.4518 (18)
S1—C1	1.7593 (14)	C9—C11	1.4936 (19)
O3—C7	1.3265 (16)	C10—H10A	0.9800
O3—H3O	0.8400	C10—H10B	0.9800
O4—C9	1.2509 (16)	C10—H10C	0.9800
N1—C8	1.4373 (16)	C11—C12	1.3966 (19)
N1—C10	1.4753 (18)	C11—C16	1.3969 (19)
C1—C2	1.3896 (19)	C12—C13	1.391 (2)
C1—C6	1.4011 (19)	C12—H12	0.9500
C2—C3	1.386 (2)	C13—C14	1.390 (2)
C2—H2	0.9500	C13—H13	0.9500
C3—C4	1.388 (2)	C14—C15	1.389 (2)
C3—H3	0.9500	C14—H14	0.9500
C4—C5	1.390 (2)	C15—C16	1.388 (2)
C4—H4	0.9500	C15—H15	0.9500
C5—C6	1.3974 (19)	C16—H16	0.9500
C5—H5	0.9500		
			
O2—S1—O1	118.95 (7)	C7—C8—N1	120.26 (11)
O2—S1—N1	108.49 (7)	C7—C8—C9	120.23 (12)
O1—S1—N1	107.25 (6)	N1—C8—C9	119.51 (12)
O2—S1—C1	109.77 (7)	O4—C9—C8	119.85 (12)
O1—S1—C1	107.98 (6)	O4—C9—C11	118.55 (12)
N1—S1—C1	103.26 (6)	C8—C9—C11	121.55 (12)
C7—O3—H3O	109.5	N1—C10—H10A	109.5
C8—N1—C10	116.02 (11)	N1—C10—H10B	109.5
C8—N1—S1	114.56 (9)	H10A—C10—H10B	109.5
C10—N1—S1	118.88 (9)	N1—C10—H10C	109.5
C2—C1—C6	121.72 (13)	H10A—C10—H10C	109.5
C2—C1—S1	120.23 (11)	H10B—C10—H10C	109.5
C6—C1—S1	117.96 (10)	C12—C11—C16	119.90 (13)
C3—C2—C1	118.87 (14)	C12—C11—C9	121.08 (12)
C3—C2—H2	120.6	C16—C11—C9	118.60 (12)
C1—C2—H2	120.6	C13—C12—C11	119.51 (13)
C2—C3—C4	120.34 (14)	C13—C12—H12	120.2
C2—C3—H3	119.8	C11—C12—H12	120.2
C4—C3—H3	119.8	C14—C13—C12	120.42 (14)
C3—C4—C5	120.73 (15)	C14—C13—H13	119.8
C3—C4—H4	119.6	C12—C13—H13	119.8
C5—C4—H4	119.6	C15—C14—C13	120.07 (14)
C4—C5—C6	119.87 (15)	C15—C14—H14	120.0
C4—C5—H5	120.1	C13—C14—H14	120.0
C6—C5—H5	120.1	C16—C15—C14	119.93 (14)
C5—C6—C1	118.46 (13)	C16—C15—H15	120.0
C5—C6—C7	120.69 (13)	C14—C15—H15	120.0
C1—C6—C7	120.79 (12)	C15—C16—C11	120.14 (14)
O3—C7—C8	122.43 (12)	C15—C16—H16	119.9
O3—C7—C6	114.62 (12)	C11—C16—H16	119.9
C8—C7—C6	122.84 (12)		
			
O2—S1—N1—C8	−166.36 (9)	C1—C6—C7—C8	−13.2 (2)
O1—S1—N1—C8	63.98 (11)	O3—C7—C8—N1	175.55 (12)
C1—S1—N1—C8	−49.92 (11)	C6—C7—C8—N1	−8.6 (2)
O2—S1—N1—C10	−22.94 (13)	O3—C7—C8—C9	−4.2 (2)
O1—S1—N1—C10	−152.60 (11)	C6—C7—C8—C9	171.61 (12)
C1—S1—N1—C10	93.50 (12)	C10—N1—C8—C7	−100.95 (16)
O2—S1—C1—C2	−37.60 (14)	S1—N1—C8—C7	43.55 (16)
O1—S1—C1—C2	93.50 (12)	C10—N1—C8—C9	78.84 (16)
N1—S1—C1—C2	−153.13 (11)	S1—N1—C8—C9	−136.66 (11)
O2—S1—C1—C6	145.77 (11)	C7—C8—C9—O4	13.9 (2)
O1—S1—C1—C6	−83.13 (12)	N1—C8—C9—O4	−165.87 (13)
N1—S1—C1—C6	30.25 (12)	C7—C8—C9—C11	−163.55 (13)
C6—C1—C2—C3	0.6 (2)	N1—C8—C9—C11	16.66 (19)
S1—C1—C2—C3	−175.86 (11)	O4—C9—C11—C12	−137.23 (14)
C1—C2—C3—C4	−0.3 (2)	C8—C9—C11—C12	40.27 (19)
C2—C3—C4—C5	0.0 (2)	O4—C9—C11—C16	35.29 (19)
C3—C4—C5—C6	−0.1 (2)	C8—C9—C11—C16	−147.21 (14)
C4—C5—C6—C1	0.4 (2)	C16—C11—C12—C13	−0.6 (2)
C4—C5—C6—C7	177.71 (13)	C9—C11—C12—C13	171.87 (13)
C2—C1—C6—C5	−0.7 (2)	C11—C12—C13—C14	1.1 (2)
S1—C1—C6—C5	175.85 (10)	C12—C13—C14—C15	−0.1 (2)
C2—C1—C6—C7	−178.00 (13)	C13—C14—C15—C16	−1.4 (2)
S1—C1—C6—C7	−1.42 (18)	C14—C15—C16—C11	2.0 (2)
C5—C6—C7—O3	−14.30 (19)	C12—C11—C16—C15	−1.0 (2)
C1—C6—C7—O3	162.91 (13)	C9—C11—C16—C15	−173.59 (13)
C5—C6—C7—C8	169.55 (13)		

### Hydrogen-bond geometry (Å, °)

**Table:**

*D*—H···*A*	*D*—H	H···*A*	*D*···*A*	*D*—H···*A*
O3—H3O···O4	0.84	1.80	2.5365 (15)	146
O3—H3O···O1^i^	0.84	2.56	3.0108 (15)	115
C3—H3···O1^ii^	0.95	2.50	3.2627 (18)	138
C12—H12···N1	0.95	2.59	3.0163 (18)	107

Symmetry codes: (i) −*x*, −*y*+1, −*z*+1; (ii) −*x*, −*y*, −*z*+1.
